# Shrinking Fabrication of a Glucose‐Responsive Glucagon Microneedle Patch

**DOI:** 10.1002/advs.202203274

**Published:** 2022-08-11

**Authors:** Zejun Wang, Ruxing Fu, Xiao Han, Di Wen, Yifan Wu, Song Li, Zhen Gu

**Affiliations:** ^1^ Department of Bioengineering University of California Los Angeles CA 90095 USA; ^2^ Department of Chemistry College of Sciences Northeastern University Shenyang 110819 China; ^3^ College of Pharmaceutical Sciences Zhejiang University Hangzhou 310058 China; ^4^ Liangzhu Laboratory Zhejiang University Medical Center Sir Run Run Shaw Hospital Hangzhou 310058 China; ^5^ Jinhua Institute of Zhejiang University Jinhua 321299 China; ^6^ Department of General Surgery, Sir Run Run Shaw Hospital, School of Medicine Zhejiang University Hangzhou 310016 China

**Keywords:** 3D printing, drug delivery, glucose‐responsive, shrinking, washable

## Abstract

A microdevice that offers glucagon supplements in a safe, non‐invasive, and glucose‐responsive manner is ideal for avoiding fatal hypoglycemia consequences from insulin overdosage during daily diabetes treatment. However, mold‐assisted microfabrication of biomedical materials or devices typically needs high‐resolution laser ablation to scale down structural design. In addition, the majority of the polymeric drug delivery materials being used to fabricate devices are dissolvable or deformable in aqueous environments, which restricts washing‐based cleaning and purification procedures post shape fixation. This study leverages the design flexibility of 3D printing‐assisted mold casting and presents a shrinking microfabrication approach that allows subsequent washing procedures to remove toxic monomer residues during polymerization. The feasibility of this approach is demonstrated by developing a glucose‐responsive transdermal glucagon microneedle patch through matrix volume change‐mediated release kinetic control. Shown in the type 1 diabetic mouse model, this transdermal patch can reverse the occurrence of hypoglycemia while lowering the risk of monomer residue‐induced irritation during treatment. Freeing from the restrain of molding resolution for microstructure design, this shrinking methodology further provides an insight into post‐fabrication purifications of biomedical materials.

## Graphical abstract

The shrinking microfabrication strategy grants sufficient purification of monomer residues after polymerization and leverages dehydration shrinkage to achieve the designated device size. This approach expands the library of polymeric candidates that have less superior biocompatibility to be used for biomedical purposes. A glucose‐responsive glucagon microneedle patch is generated with this method for dealing with hypoglycemia emergencies.

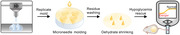

## 1 Introduction

Hypoglycemia is recognized as a life‐threatening condition that accompanies abnormally low blood glucose levels.^[^ ^1^ ^]^ It is a common side‐effect of diabetes treatment where overdosage frequently occurs during direct insulin supplements or consumption of insulin secretagogues.^[^ ^2^ ^]^ Mild symptoms such as difficulty concentrating, sweating, anxiety, and vision changes could be resolved through carbohydrate intake. However, severe episodes require immediate assistance in responding to fatal consequences such as sudden unconsciousness and coma.^[^ ^3^ ^]^ Thus, an act‐on‐need external glucagon supplementary strategy is highly demanding in these unpredictable hypoglycemic emergencies.^[^ ^4^ ^]^ Considering diabetes treatment is a daily task, frequent occurrence of hypoglycemia is expected that calls for repeated administration of glucagon.^[^ ^5^ ^]^ Therefore, two major concerns regarding the delivery system in this scenario need to be fulfilled: material bioavailability and fabrication simplification of formulations and devices.

Given the desirable patient compliance and accessibility of the skin, transdermal drug delivery approaches have been regarded as a preferred alternative to hypodermic injections.^[^ ^6^ ^]^ Various physical and chemical techniques for enhancing the transdermal penetration efficacy have facilitated the delivery of macromolecular drugs.^[^ ^7^ ^]^ Microneedle array patches are among the simplest, non‐invasive means that can be designed with versatile materials, properties, and functions.^[^ ^8^ ^]^ In particular, the use of microneedle array patches could gain further control over the diffusion kinetics of the drugs.^[^ ^9^ ^]^ To achieve higher drug loading capacity and smart control over the release kinetics, in situ polymerization of functional copolymers are ideal for polymeric microneedles design. A series of glucose‐responsive glucagon microneedle patches have been developed for this purpose.^[^ ^10^ ^]^ However, the microneedle tips escort drugs to the destination by creating micro‐channels in the skin, which in turn expose the skin to toxic chemicals such as diffusive monomers if the material is not thoroughly cleaned, limiting matrix design to only a few biocompatible polymers or copolymers free of toxic monomer residues.^[^ ^11^ ^]^ Additionally, the drug release mechanism mainly utilizes the solvent dissolvable or deformable properties of the polymeric matrixes, which hinders the pre‐administration of the purification process.^[^ ^12^ ^]^ Despite the different purposes of the microneedle, mold‐assisted fabrication is the dominant method. The size and shape qualities of the mold cavities govern the final appearance delicacy of the microneedle thus requiring advanced photolithography facilities and soft lithography techniques to achieve fine structural resolution.^[^ ^13^ ^]^

Towards these goals, here we describe the shrinking fabrication of a glucose‐responsive glucagon microneedle patch using PDMS molds cast from 3D printed microneedle masters that could tolerate post‐polymerization washing. The glucose‐responsive property of the encapsulation matrix in partner with the follow‐up washing step could prevent physical and bioactivity loss of the preloaded glucagon while eliminating toxic monomer residues to a safe level. As demonstrated in the type 1 diabetic mice model, this patch has shown the ability to counteract dangerously low plasma glucose levels.

## 2 Results

### 2.1 Shrinking Fabrication of the Microneedle Patch

**Figure 1:**
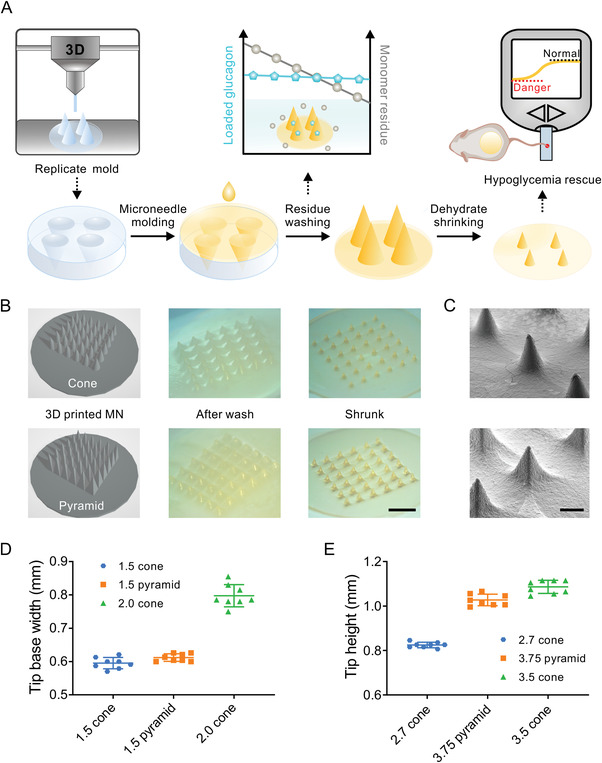
Shrinking fabrication of the microneedle patch. A) Schematic illustration of the shrinking fabrication process. B) 3D design drawing and images of the 1.5 cone (top panel) and 1.5 pyramid‐shaped (bottom panel) MN patch before (middle) and after shrinkage (right). Scale bar, 3 mm. C) SEM images of the 1.5 cone (top) and 1.5 pyramid (bottom) shaped MN patch. Scale bar, 500 µm. The D) tip base width and E) needle height change of the different MNs after full shrinkage. Data points are means ± SD (*n* = 8).

In terms of the dilemma towards purification‐induced structure distortion after polymeric microneedle shape fixation, the shrinking fabrication concept focuses on switching the order of these two procedures. Briefly, the mold‐casting process is utilized to first generate a relatively large preliminary microneedle gel with a determined shape and arrangement followed by subsequent dehydration to finalize size miniaturization (**Figure** **1**A). The initial gel‐like status allows facile control over the microneedle shape, molding process, and purification while the shrinking step assures retained shape details and mechanical strength of the miniaturized microneedle.

To assess the structural preservation accuracy throughout the shrinking fabrication, cone‐shaped or pyramid‐shaped microneedle masters were 3D printed with a commercial 3D printer for generating replicating PDMS molds (Figure 1B and Figure S1, Supporting Information). The enlarged “millimeter‐sized” molds allow fast casting of the precooled polymeric solutions simply by pipetting and sonication. The 5‐min polymerization process takes place in the 37 °C incubator, circumventing the underlying damage to the cargo protein during heat or UV‐induced shape fixation. After purification, the gel‐like microneedle was mounted on the transparent polycarbonate film and half‐sealed for slow dehydration until the microneedle base detaches from the polycarbonate film.

During the shrinking process, the base of the patch can decrease in thickness while retaining its original size and shape due to the stabilization of the polycarbonate film. Similarly, the center distance between each microneedle tip is maintained but the microneedle tip itself contracts both horizontally and vertically to obtain a uniform predefined shape (Figure 1B,C). This shrinking pattern is reflected in all three groups of MNs evaluated: 1.5 mm tip base width cone‐shaped MN (1.5 cone), 1.5 mm tip base width pyramid‐shaped MN (1.5 pyramid), and 2.0 mm tip base width cone‐shaped MN (2 cone). The tip base width (*w*) and height (*h*) after shrinkage was measured for 1.5 cone (w 39.7%, h 30.5%), 1.5 pyramid (w 40.8%, h 27.4%), and 2 cone (w 39.9%, h 31.0%) shaped microneedle, respectively (Figure 1D,E). Generally, cone and pyramid‐shaped microneedles share a similar contraction pattern where the shrinkage rate is higher in the vertical direction. In consideration of the ease of fabrication and uniform shrinkage, the 6 × 6 mm cone‐shaped microneedle mold (1.5 mm width × 2.7 mm height) that could shrink into a 0.6 × 0.8 mm microneedle was adopted for the subsequent experiments. In correlation with the glucose‐induced volume shifting release mechanism, glucose‐dependent swelling of the GRS glucagon MN patch was observed when re‐immersed in glucose‐containing PBS solutions (Figure S2, Supporting Information).

### 2.2 Microneedle Matrix Design for Glucose‐Responsive Glucagon Release

**Figure 2:**
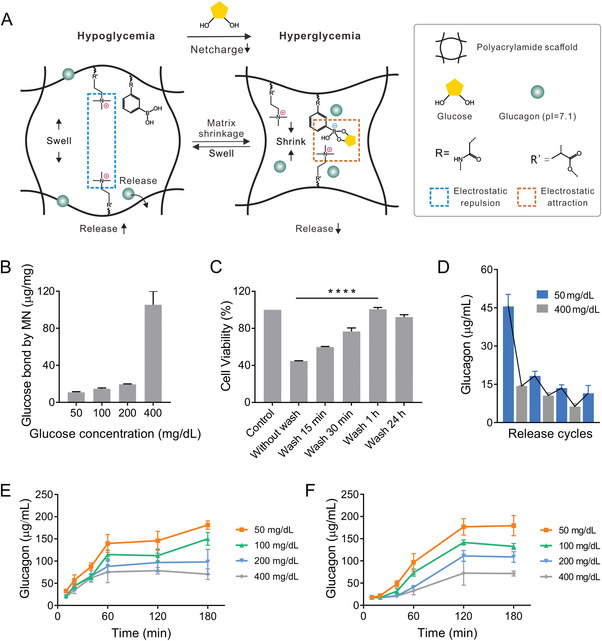
Mechanism and in vitro performance of the GRS glucagon delivery system. A) The schematic illustration shows that in hypoglycemic conditions, the expanded cavities in the cationic polyacrylamide matrix promote the release of glucagon. As glucose level elevates, the negatively charged glucose‐boronate complexes neutralize the surrounding repulsion force to induce the shrinkage of the polymer and slow down glucagon diffusion. B) The concentration‐dependent glucose‐binding ability of the washable glucose‐responsive glucagon MN. C) The prolonged prewash of the polymeric matrix increases cell viability. D) Pulsatile glucagon release by alternating glucose concentrations between 50 and 400 mg dL^−1^ for three consecutive cycles. Accumulated glucagon release from the GRS glucagon MN patch E) before and F) after 1‐h of prewash in varying glucose concentrations at 37 °C, pH 7.4. Data points are means ± SD (*n* = 3). ****p < 0.0001.

To pursue efficient delivery, controlling the release of glucagon is equally essential in the purification step and during treatment. Unlike its counter‐regulatory partner insulin, which exhibits a negative charge at physiological pH, glucagon has a pI value of ≈7.1,^[^ ^14^ ^]^ making it a near‐neutral charge state in a physiological environment. Therefore, responsive release design relies mainly on the swelling and shrinking transformation of the matrix at designated glucose concentrations. Here, the glucose‐responsive shrinking (GRS) matrix is the copolymer of acrylamide, [2‐(methacryloyloxy)ethyl]trimethylammonium (MAETAC), and 3‐(acrylamido)phenylboronic acid (APBA), with bisacrylamide as the crosslinker, ammonium persulfate (APS), and *N, N, N', N'*‐tetramethylethylenediamine (TEMED) as the catalyst. This combination ensures mild and fast polymerization, washing tolerable, and exhibits a desirable shrinking ratio and mechanical rigidity for skin penetration. Cationic polyacrylamide (CPAM) acts as the scaffold to encapsulate glucagon peptide, and APBA serves as the glucose‐responsive charge tuning unit. The mechanism of glucagon delivery in response to low glucose levels is attributed to the net charge shift from cationic to neutral of the CPAM/APBA polymeric network (**Figure** **2**A). When hypoglycemia occurs, the repulsive cationic matrix can facilitate the release of glucagon for correcting dangerously low blood glucose. As plasma glucose increases, the elevated number of glucose molecules binding to APBA can contribute more negative charges to promote electrostatic attraction (Figure 2B). This results in a “seize tight” matrix status, restricting excess glucagon release.

Leveraging the glucose concentration‐sensitive volumetric alteration behavior of the matrix, purification is performed in phosphate‐buffered saline (PBS) washing buffer supplemented with 400 mg dL^−1^ glucose to intercept glucagon loss. Purification time length versus matrix cytotoxicity towards mouse fibroblast L929 cells was evaluated (Figure 2C) with overnight incubation. MTT results showed that the cell viability increased from ≈45% to ≈77% after 30 min of immersion in the washing solution. The matrix showed negligible cytotoxicity after 1 h of purification, during which a 2 wt% leach of the total glucagon was observed (Figure S3, Supporting Information). As purification time was prolonged to 24 h, the percentage of glucagon loss doubled. To minimize the loss of payloads, 1‐h washing time was adopted for the remaining experiments.

The pulsatile glucagon release profile was characterized by alternating incubation of the polymeric matrix in the low glucose level (50 mg dL^−1^) and high glucose level (400 mg dL^−1^) PBS solutions. Three cycles of glucose‐responsive release (Figure 2D) were demonstrated with a gradual drop in release amount after each cycle of consumption. Before and after purification, a similar negative correlation between glucose concentration and glucagon releasing kinetics was observed in vitro (Figure 2E,F), thus purification does not perturb the glucose‐responsive function.

### 2.3 Purification and Glucose‐Responsive Evaluation of the GRS Glucagon MN Patch In Vivo

**Figure 3:**
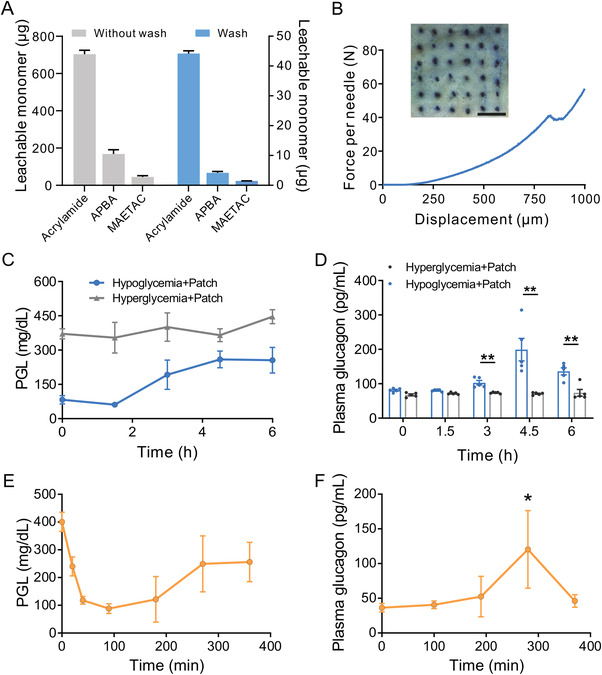
Evaluation of the purification and in vivo glucose‐responsive performance of the GRS glucagon MN patch. A) HPLC quantification of leachable monomers before and after 1‐h purification. B) Mechanical performance of the GRS glucagon MN patch. MN insertion (inset photo) was evaluated by staining mouse dorsum skin with trypan blue after being treated with the shrinking MN‐array patch. Scale bar, 3 mm. C) PGLs and D) plasma glucagon concentrations in diabetic mice after being treated with the patch (glucagon dose: 40 mg kg^−1^) in a hyperglycemic state and a hypoglycemic state (induced by 15‐h overnight fasting and a subcutaneous injection of 75 µg kg^−1^ insulin), respectively. E) PGLs and F) plasma glucagon concentrations in diabetic mice treated with the patch (glucagon dose: 40 mg kg^−1^) which was challenged with an i.p. injection of 3 mg kg^−1^ insulin 1‐h post‐patch administration. Data represents means ± SD (*n* = 5). Statistical significance was determined by a two‐tailed Student's *t*‐test. *p < 0.05, **p < 0.01.

Prior to deploying the GRS glucagon MN patch in vivo, we looked into the clearance efficacy of the residue monomers during 1‐h purification. The as‐prepared patches were immersed in PBS for 3 h to collect leachable monomers which were quantified using high‐performance liquid chromatography (HPLC, Table S1, Supporting Information). Upon purification, the total amount of leachable acrylamide, APBA, and MAETAC exhibited reductions of 16, 40, and 31 folds, respectively (**Figure** **3**A, Tables S2 and S3, Supporting Information). The detected amount of the leachable monomers shown in Table S3, Supporting Information is well within the safety range in the reported database (https://pubchem.ncbi.nlm.nih.gov/). Additionally, hematoxylin and eosin (H&E) staining of the mice skin treated with the pre‐washed or post‐washed GRS glucagon MN patch for 24 h was evaluated, respectively. The pre‐washed group experienced increased dermal thickness and notable inflammation on day 3 compared to the post‐washed group, reaffirming the purification necessity for skin‐contacting devices. For both groups, inflammation if any at the administration sites was mostly healed 1 week after withdrawal (Figure S4, Supporting Information).

Despite the initial gel‐like structure, fully shrunken GRS glucagon MN patches exhibit superior mechanical strength compared with various reported MNs.^[^ ^15^ ^]^ The average fracture force exceeds 40 N per needle, which allows for easy skin penetration in the subsequent studies (Figure 3B and Figure S5, Supporting Information). In vivo glucose‐responsiveness was assessed by characterizing the release profile of the GRS glucagon MN patch on two groups of streptozotocin‐induced type 1 diabetic mice: one with hyperglycemic condition (untreated) and one with hypoglycemic condition (treated with 15‐h fasting and a subcutaneous injection of 75 µg kg^−1^ insulin). Plasma glucagon quantification with an enzyme‐linked immunosorbent assay (ELISA) displayed a higher glucagon release in the hypoglycemia group starting at 3‐h post‐administration, consistent with the subsequent sustained normoglycemia levels. By contrast, the untreated diabetic mice group showed no notable rise in plasma glucagon level which is also reflected in the steady plasma glucose levels (PGLs) (Figure 3C‐D). We further performed an intraperitoneal insulin tolerance test (IPITT) with an i.p. injection of 3 mg kg^−1^ insulin 1 h post‐GRS glucagon MN patch administration. In response to the insulin challenge, the PGLs decreased and approached hypoglycemia in 1.5 h (Figure 3E). Accordingly, a gradual increase in the plasma glucagon level was observed following the drop in PGLs (Figure 3F). Taken together, the GRS glucagon MN patch displayed a glucose‐regulatory release pattern.

### 2.4 In Vivo Plasma Glucose Level Control Performance of the GRS Glucagon MN Patch

**Figure 4:**
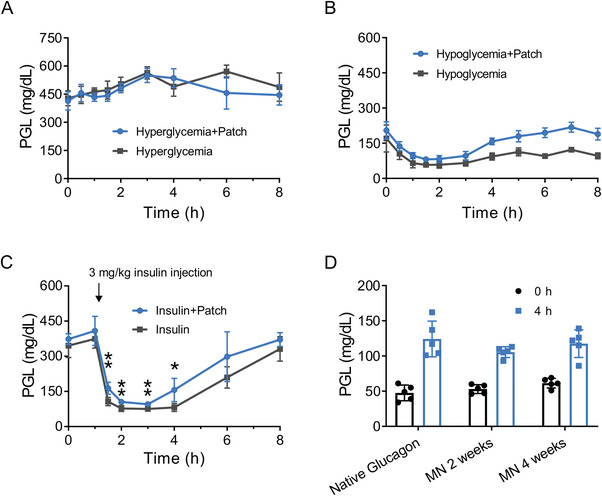
Safeguard and preservation evaluation of the GRS glucagon MN patch. A) PGLs of diabetic mice treated with the patch (glucagon dose: 40 mg kg^−1^) versus control mice (no treatment). B) PGLs of diabetic mice in a hypoglycemic state (induced by overnight 15‐h fasting with 75 µg kg^−1^ insulin injection) with and without patch administration (glucagon dose: 40 mg kg^−1^). C) PGLs recovery after 3 mg kg^−1^ insulin injection 1 h (indicated by arrow) post glucagon patch administration (glucagon dose: 40 mg kg^−1^). D) PGL increasing ability of the glucagon extracted from the MN patch stored at room temperature (for 2 or 4 weeks) on diabetic mice in a hypoglycemic state (induced by subcutaneous injection of 3 mg kg^−1^ insulin). PGLs of the hypoglycemia mice were monitored before and 4 h after the subcutaneous injection, respectively. Data represents means ± SD (*n* = 5). Statistical significance was determined by a two‐tailed Student's t‐test. *p < 0.05, **p < 0.01.

In vivo glycemic regulation ability of the GRS glucagon MN patch to lower hypoglycemia risks was further assessed with a streptozotocin (STZ) ‐induced insulin‐deficient diabetic mouse model. The hypoglycemia‐activated glucagon booster release from the GRS glucagon MN patch was validated on two groups of diabetic mice: one exhibiting hyperglycemic PGLs (untreated) and one undergoing hypoglycemic PGLs (treated with 15 h‐fasting and a subcutaneous injection of 75 µg kg^−1^ insulin). Monitored with a glucometer, the GRS glucagon MN patch administered hyperglycemia group showed a negligible difference in PGLs compared to the hyperglycemia control group (**Figure** **4**A). By contrast, under hypoglycemia status, the GRS glucagon MN patch treated group showed notably higher PGLs, which reached and maintained in the normal range 3‐h post‐administration (Figure 4B). Additionally, when the hyperglycemia mice were subcutaneously injected with an over‐dosage of insulin (3 mg kg^−1^), the GRS glucagon MN patch could prevent PGLs from dropping below 50 mg dL^−1^ while gaining a more rapid PGL recovery 3 h post insulin challenge (Figure 4C). This hypoglycemia reversing performance is also reflected in the remarkably reduced hypoglycemic index (defined by the difference between the initial and nadir PGL readings divided by the time spent to reach nadir) average of 2.6, compared with 4.5 of the non‐treated group (Figure S6, Supporting Information).

The mild GRS glucagon MN patch preparation process ensured that the encapsulated glucagon exhibited a comparable hyperglycaemic function as freshly dissolved glucagon when released in the diabetic mice (Figure 4D). Mass spectrum analysis also revealed that the glucagon molecule structure was intact after the microneedle fabrication and purification process (Figure S7, Supporting Information). Moreover, microneedle‐sheltered glucagon preserved at room temperature displayed a better hyperglycaemic effect after release compared to the native glucagon solution stored at 4 °C. This stabilization effect of the microneedle patch was observed to be at least 4 weeks (Figure 4D and Figure S8, Supporting Information).

## 3 Discussion and Conclusion

Gaining safety control over polymeric devices aimed at clinical applications is of critical importance. Herein, the challenge of eliminating harmful residues from solution‐deformable polymeric device modules without disrupting their defined structure is addressed with a shrinking strategy. Leveraging the volume difference and dehydration pattern of polymeric matrixes in response to the presence of aqueous solution, we advanced the purification step ahead of shape fixation in the micro‐molding process. The swelling status enables fast diffusion of leachable monomers during washing. Dehydrated condition finalizes shaping to the desired miniaturized size. The incorporation of stimuli‐responsive release could effectively restrict cargo loss in the purification stage. The proof of concept study based on the glucose‐responsive glucagon‐supplementing microneedle patch fabrication achieved anticipatory hypoglycemia reversing outcome in the diabetic mice with negligible safety concerns as well as prolonged functional stability of the loaded glucagon.

With this purification‐guaranteed fabrication concept, an augmented library of polymeric materials and molecules that have less superior biocompatibility could be adopted for microneedle fabrication to achieve desirable matrix functionalization. Future exploration of the correlation between the solvent ratio of various polymers with dehydration properties in the swelling state with the final shrinkage rate could entrust scaling with increased programmability.^[^ ^16^ ^]^ We foresee the expanding applications of the approach in microfluidics, soft robotics, wearable sensors, and various biomedical purposes that involve direct contact with tissues.

## 4 Experimental Section

### Materials

All chemicals were purchased from Sigma‐Aldrich unless otherwise specified and were used as received. 3‐(acrylamido)phenylboronic acid was purchased from Boron Molecular (catalog number BM1195). Glucagon, D‐glucose, tetramethylethylenediamine, bis‐acrylamide, and ammonium persulfate were purchased from Fisher Scientific (catalog number 50‐751‐6116, D16‐3, PI17919, BP171‐25, AC401160100).

### Cell Lines

The L929 cell line was purchased from ATCC. Cells were cultured in EMEM (BD Biosciences) with 10% horse serum and 100 U mL^−1^ penicillin, 100 µg mL^−1^ streptomycin (Thermo Fisher Scientific) at 37 °C in 5% CO_2_.

### 3D Printing

The microneedle masters were designed with Autodesk 3ds Max and printed with a Formlabs Form 3 3D printer using Clear Resin (Formlabs) as the printing material. The layer thickness is 50 µm. The prints were washed with isopropanol for 15 min, then cured at 60 °C for 15 min in Form Cure before use.

### Microneedle Fabrication

The microneedle was prepared by dissolving acrylamide (40 mg), glucagon (10 mg), 3‐(acrylamido)phenylboronic acid (15 mg), 75 wt% [2‐(methacryloyloxy)ethyl]trimethylammonium chloride solution (20 µL) in 400 µL 0.75% (wt) *N,N'*‐methylenebisacrylamide in the above mentioned order for optimized solubility. The mixture was then placed on ice before adding 10% ammonium persulfate (38 µL) and 10% tetramethylethylenediamine (38 µL). Then, the solution was instantly deposited by pipette onto the microneedle mold surface (operated on ice) and bubbles could be eliminated by sonication. The mold was incubated at 37 °C for 5 min to complete crosslinking and was dipped in 10 mL pH 7.4 PBS wash solution (containing 400 mg dL^−1^ glucose), where the microneedle could be easily detached from the mold and was immersed for 1 h at room temperature to remove the unreacted monomers. After purification, the patch was placed on a 0.01‐inch‐thick, transparent polycarbonate film (McMaster‐Carr) to be dried overnight at room temperature in a half‐covered petri dish. The fully dehydrated microneedle patch self‐detached from the polycarbonate film and was stored in a sealed container at room temperature for further study.

### Mechanical Strength Test

The mechanical strength of the MNs was assessed by an Instron 5564 tensile compression machine. The initial gauge was set to 2 mm between the MN tips and the stainless‐steel plate, with 100 N as the load cell capacity. The speed of the top stainless‐steel plate movement towards the MNs was 0.25 mm min^−1^. The failure force of the MNs was recorded when the needles began to buckle.

### In Vitro Glucose Binding Study

The samples were immersed in 1 mL PBS containing various concentrations of glucose (50, 100, 200, and 400 mg dL^−1^) and incubated at 37 °C with gentle shaking for 3 h. The glucose concentration was measured with a Clarity GL2PLUS glucose meter (Clarity Diagnostics), which was calibrated using a glucose standard curve in advance.

### In Vitro Release Studies

To evaluate the glucose responsiveness of the glucagon formulation, ≈10 mg prewashed or washed samples were incubated in 1 mL of PBS solution (pH 7.4) with various glucose concentrations (50, 100, 200, and 400 mg dL^−1^) at 37 °C with gentle shaking (150 rpm). At predetermined time points, 10 µL of the supernatant was collected, and the released insulin or glucagon was quantified using a Coomassie (Bradford) protein assay (Thermo Fisher Scientific) in a 96 well plate (100 µL well^−1^). The absorbance was detected at 595 nm on the Infinite 200 Pro multimode plate reader (Tecan Group), and the concentration was calculated with glucagon (8–1000 µg mL^−1^) standard. Pulsatile release of glucagon was measured using the same procedure by alternating the glucose concentrations between 50 and 400 mg dL^−1^ in the PBS solution every 30 min of incubation.

### Insulin Solution

20 mg mL^−1^ insulin stock solution was prepared by dissolving human recombinant insulin in 0.1 M HCl. 1 mg mL^−1^ and 15 µg mL^−1^ insulin injection solutions were generated by diluting the stock solution with pH 7.4 PBS buffer.

### Animal Experiments

All animal experiments were performed in compliance with an animal study protocol approved by the Institutional Animal Care and Use Committee at University of California, Los Angeles (ARC‐2018‐062). The in vivo performance of the patches was evaluated on streptozotocin‐induced adult diabetic mice (male C57BL/6J, age 7–8 weeks; Jackson Laboratory). For MN insertion, a medical tape (3M Tegaderm Transparent Film) was adhered to the base of the patch for better immobilization before applying to the back of the mouse and pressed firmly for 15 s. To avoid movement, the mice were anesthetized with isoflurane during the application of the patch. After insertion, the PGLs were recorded with an Accu‐Chek Aviva (Roche Diabetes Care, Inc.) glucometer.

### Plasma Glucagon Level Measurement

The plasma glucagon level was measured by collecting 25 µL of plasma at different time points, which was stored at −20 °C until measurement using human glucagon (catalog number EHGCG) enzyme‐linked immunosorbent assay (ELISA) kit (Thermo Fisher Scientific) following the manufacturer's instruction.

### H&E Staining Experiment

The glucagon MN patches were applied to the shaved backs of the mice for 24 h. On days 3 and 7 after MN removal, mice were euthanized, pieces of skin from the treated sites were harvested and fixed in 4% formaldehyde for 24 h before H&E staining by Translational Pathology Core Laboratory at Pathology & Laboratory Medicine, UCLA. Histopathology images were acquired on Eclipse Ti2 fluorescence microscopy (Nikon).

### Quantification of Leachable Monomers

To analyze the washing efficacy of the monomers from the microneedles, the number of leachable monomers before and after washing was obtained by HPLC (Waters Alliance e2695 Separations Module with 2998 PDA Detector). Briefly, the microneedles with or without wash were incubated in 2 mL PBS (pH 7.4) at 37 °C for 24 h. The released monomers in the supernatant were measured via an XBridge BEH C18 4.6 × 100 mm column with an injection volume of 10 µL. The detailed gradient HPLC method is provided in the tables in Supporting Information and each sample was measured three times. Standard solutions of acrylamide, APBA, and MAETAC with varying concentrations were prepared by dissolving each monomer in PBS.

### Mass Spectrum Analysis

Native glucagon and glucagon released from the prepared MN patches in PBS (pH 7.4) at 4 °C for 24 h were detected using an Agilent 6530 Accurate‐Mass Q‐TOF LC/MS system.

## Conflict of Interest

Z.G. is a scientific co‐founder of ZCapsule Inc. and Zenomics Inc.

## Author contributions

The study is performed under the supervision of Z.G. and S.L. Z.W. and Z.G. are responsible for the conception of the study. Z.W., R.F., X.H., D.W., and Y.W. performed the experiments and collected data. Z.W. analyzed the data and co‐wrote the manuscript with Z.G. and S.L.

## Supporting information

Supporting Information

## Data Availability

The data that support the findings of this study are available in the supplementary material of this article.
