# Supplementary material for: Shrinking Fabrication of a Glucose‐Responsive Glucagon Microneedle Patch
Source: Adv Sci (Weinh). 2022 Aug 11;9(28):2203274. doi: 10.1002/advs.202203274 (PMC9534970; doi:10.1002/advs.202203274)
Supplement: Supplementary file 1 — Supporting Information [file ADVS-9-2203274-s001.pdf]

ADVANCED SCIENCE
Open Access

Supporting Information

for *Adv. Sci.*, DOI 10.1002/advs.202203274

Shrinking Fabrication of a Glucose-Responsive Glucagon Microneedle Patch

*Zejun Wang, Ruxing Fu, Xiao Han, Di Wen, Yifan Wu, Song Li and Zhen Gu**

Supporting Information

Shrinking fabrication of a glucose-responsive glucagon microneedle patch

*Zejun Wang, Ruxing Fu, Xiao Han, Di Wen, Yifan Wu, Song Li, Zhen Gu**

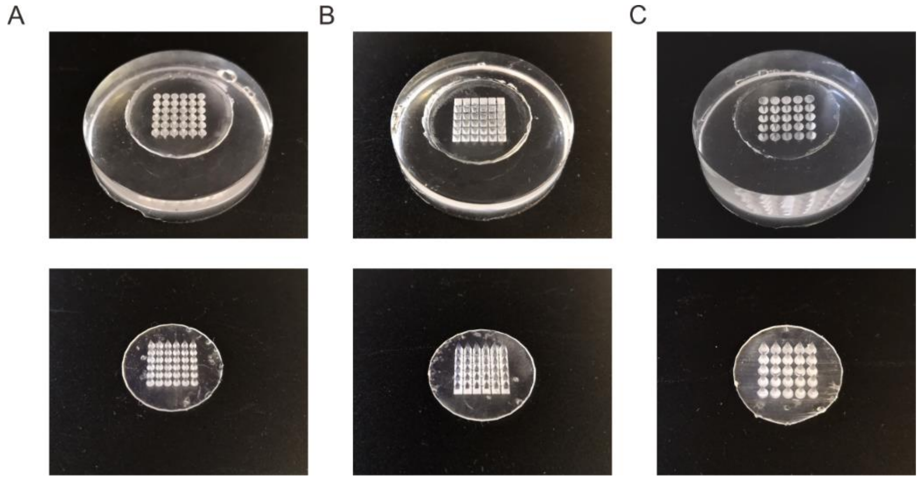

Figure S1. PDMS replicating mold (upper panel) of (A) 1.5 cone (6×6, 1.5 mm width × 2.7 mm height), (B) 1.5 pyramid (6×6, 1.5 mm width × 3.75 mm height), and (C) 2 cone (5×5, 2.0 mm width × 3.5 mm height) MN patch generated from the 3D printed MN masters (lower panel).

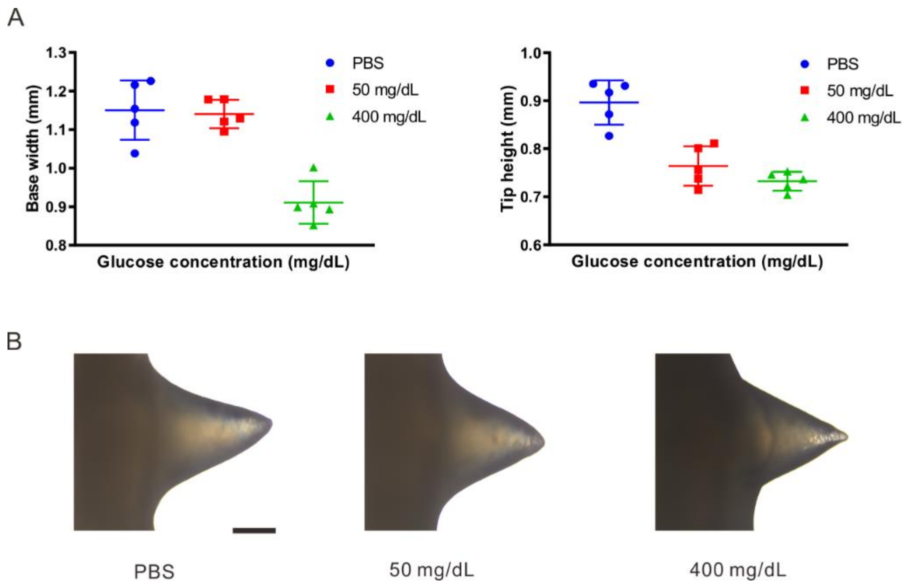

Figure S2. Glucose-dependent swelling evaluation (A) and representative images (B) of the 1.5 cone MN tips post-incubated in PBS supplemented with varying glucose concentrations for 3 h. Data are presented as means ± SD (n = 5). Scale bar, 300 μm.

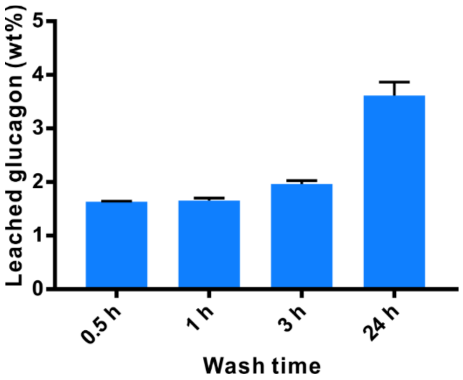

Figure S3. The amount of glucagon leached from the GRS glucagon MN patch during different washing time lengths. Data are presented as means ± SD (n = 3).

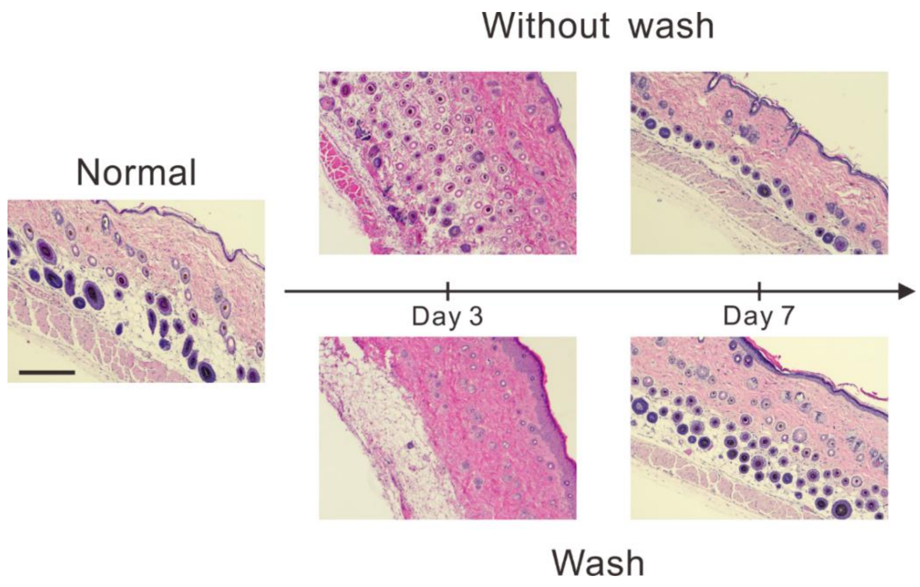

Figure S4. Hematoxylin and eosin (H&E) staining results. GRS glucagon MN patch with or without wash was applied on mice skin for 24 h, respectively. The treated parts of the skin were harvested 3 and 7 days post MN removal. Scale bar, 250 μm.

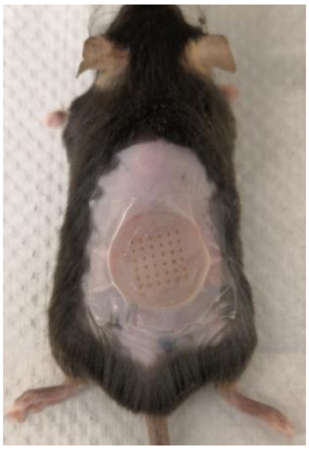

Figure S5. Representative image of the GRS glucagon MN patch placed on the mouse dorsum skin.

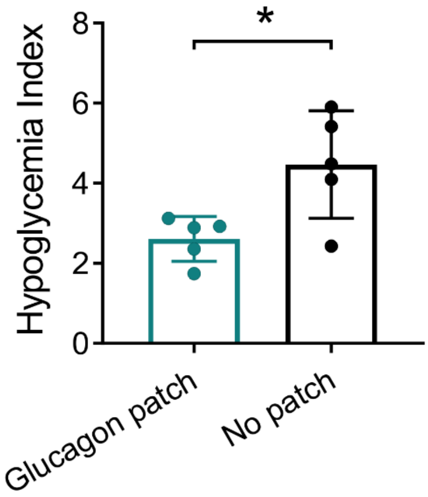

Figure S6. Hypoglycemia index of the insulin challenged diabetic mice with or without GRS glucagon MN patch treatment. Data are presented as means ± SD (*n* = 5).

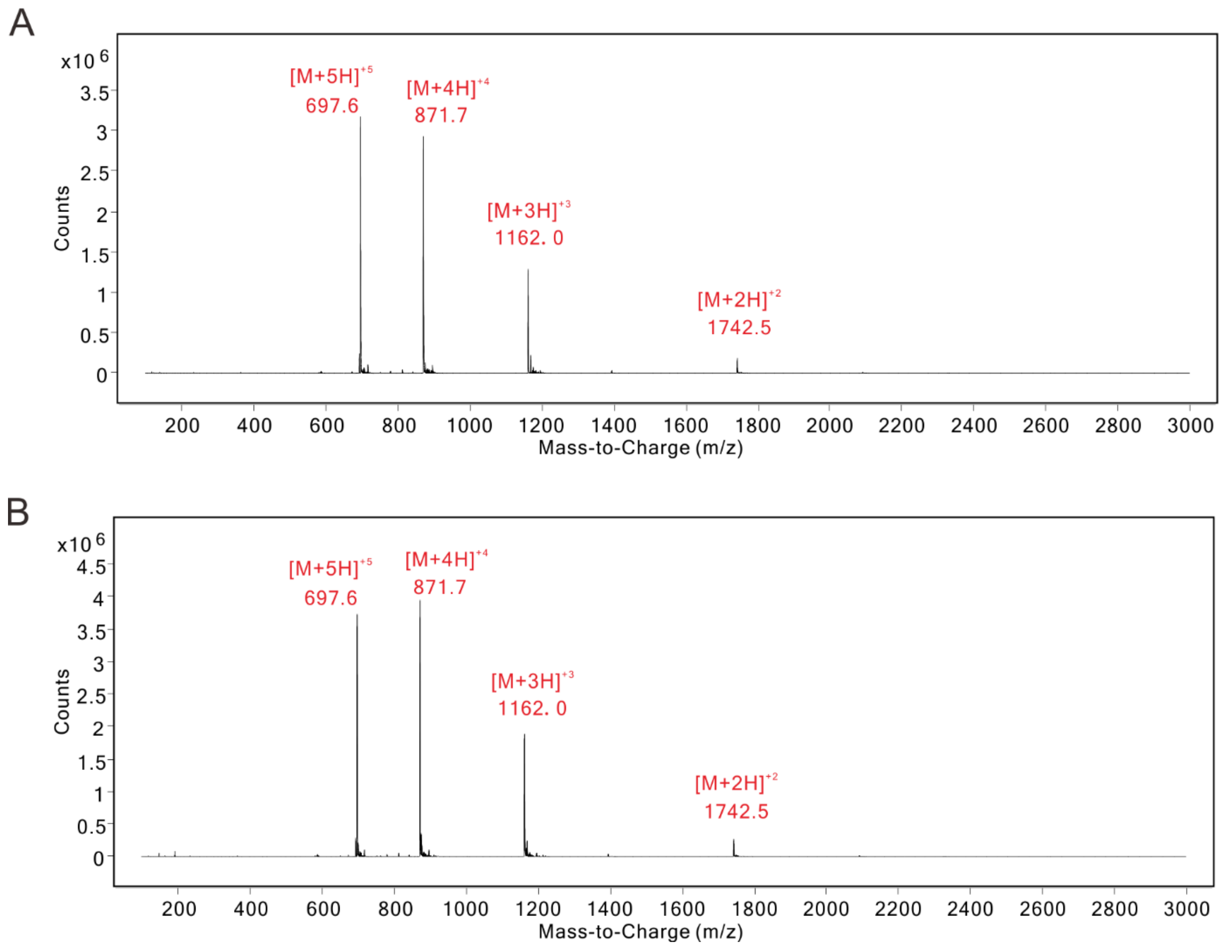

Figure S7. The mass spectrum analysis of the (A) native glucagon and (B) glucagon released from the GRS glucagon MN patch.

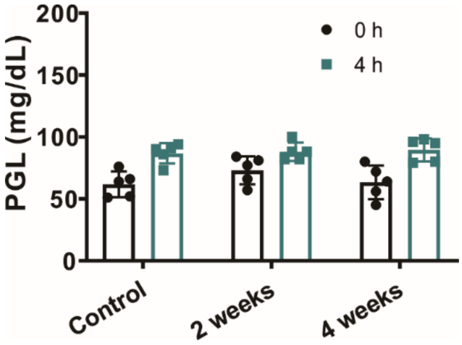

Figure S8. Hyperglycemic effects of the non-treated (control) group and groups treated with native glucagon PBS solution after 2 or 4-week storage at 4 °C. PGLs of the hypoglycemia mice were monitored before and 4 hours after the subcutaneous injection, respectively. Data are presented as means ± SD (*n* = 5).

Supplementary Table 1. Gradient HPLC method for acrylamide, APBA, and MAETAC.

Time (min)	Mobile phase A (%)	Mobile phase B (%)	Flow (mL/min)
0.00	95.0	5.0	1
4.50	70.0	30.0	1
5.50	70.0	30.0	1
8.00	50.0	50.0	1
8.10	5.0	95.0	1
9.00	5.0	95.0	1
9.10	95.0	5.0	1
10.00	95.0	5.0	1

A: Water with 0.1% formic acid (v/v)

B: Acetonitrile with 0.1% formic acid (v/v)

Supplementary Table 2. Leachable monomers from the 6×6 GRS glucagon MN patch without the 1-hour washing procedure.

Monomer	Leachable amount (μg)	wt% per patch
Acrylamide	703.88 ± 18.29	7.38 ± 0.19
APBA	168.66 ± 19.37	1.77 ± 0.20
MAETAC	45.24 ± 5.82	0.47 ± 0.06

Supplementary Table 3. Leachable monomers from the 6×6 GRS glucagon MN patch with the 1-hour washing procedure.

Monomer	Leachable amount (µg)	wt% per patch
Acrylamide	44.17 ± 0.82	0.463 ± 0.009
APBA	4.17 ± 0.41	0.044 ± 0.004
MAETAC	1.46 ± 0.02	<0.015
